# A novel physical hurdle technology by combining plasma-activated water and ultrasound for long-term stored shiitake mushrooms

**DOI:** 10.1016/j.ultsonch.2026.107840

**Published:** 2026-04-01

**Authors:** Jun Shao, Xue Huang, Yanyin Guo, Lu Sun, LuJun Zhang, Jie Li, Xinhe Zhao, Zitong Zhao

**Affiliations:** a School of Agricultural Engineering and Food Science, Shandong University of Technology, Zibo 255000, China; b Futaste Pharmaceutical Co., Ltd., Yucheng 251200, China; c Binzhou Polytechnic, Binzhou, Shandong 256603, China; d Shandong Qihe Bio Technology Co., Ltd, Zibo, Shandong 255000, China; e School of horticulture, Ludong University, Yantai 264025, Shandong Province, China

**Keywords:** Plasma-activatedwater treatment, Ultrasound treatment, Postharvest quality, Combined treatment, Shiitake mushrooms

## Abstract

The shiitake mushrooms are perishable due to microbial infection and water loss during storage. This study investigated the effect of plasma-activated water (PAW) combined with ultrasound (US) treatment to microbes and the postharvest quality of shiitake mushrooms. For combined treatments, fresh shiitake mushrooms were soaked in PAW for 10 min while being simultaneously sonicated at 110, 220, or 330 W for 10 min (PAW-US_110_, PAW-US_220_, and PAW-US_330_, respectively). The results unveiled that the PAW combined with US (PAW-US) treatment significantly reduced nearly 0.85–1.09 log natural microorganisms at 14 d of storage. The results showed that PAW-US treatment contributed to suppressing the respiration rate, inhibiting softening and browning, enhancing antioxidant capacity, and maintaining membrane integrity. Among all the groups, PAW-US_220_ treatment yielded the most pronounced effects, highlighting the potential of this hurdle technology for postharvest preservation of shiitake mushrooms.

## Graphical abstract

**Figure:**
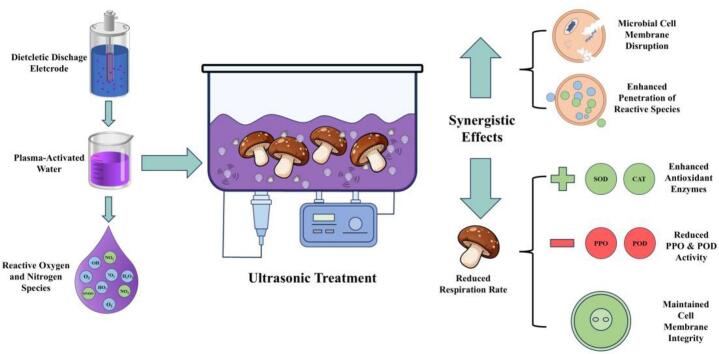

## 1 Introduction

Shiitake mushrooms (*Lentinus edodes*) have become an important economic crop worldwide due to their exceptional nutritional value, medicinal properties, and unique flavor [1]. However, they lack a protective cuticle and have high moisture content, making them susceptible to mechanical damage and microbial spoilage. Postharvest issues such as browning, softening, rotting, flavor loss, and microbial contamination significantly shorten their shelf life and deteriorate their sensory and commercial value [2].

Various preservation techniques have been explored to reduce postharvest losses and regulate physiological and metabolic processes in shiitake mushrooms. While drying and refrigeration are commonly used, excessive heat or cold may degrade nutritional and physical characteristics [3]. Physical preservation methods, such as atmosphere packaging, irradiation, and pulsed electric fields, have also been applied, but challenges such as off-flavors, high cost, and nutrient losses remain [4]. Chemical methods, including those using antibacterial agents, ozone (O_3_), and edible coatings, offer limited effectiveness and pose food safety concerns due to potential chemical residues [5]. Therefore, there is an urgent need for a safe, efficient, cost-effective, and eco-friendly preservation method that can delay mushroom senescence, reduce postharvest losses, and enhance commercial value.

Cold plasma, generated by applying electrical energy to gases such as argon, helium, oxygen, nitrogen, or air, produces high-energy electrons while keeping most other particles (gas atoms, ions, and molecules) in a low-energy state, resulting in low-temperature plasma [6]. This plasma contains reactive nitrogen species (RNS), reactive oxygen species (ROS), free electrons, ions, and ultraviolet photons. These species damage microbial lipids, proteins, DNA, and cell membranes through oxidative stress, leading to cell death. Compared with traditional sterilization methods, cold plasma offers rapid microbial inactivation at room temperature without chemical usage, thus significantly extending the food shelf life and improving food safety [7]. However, cold plasma treatment still faces challenges in terms of uniformity and other aspects. Furthermore, prolonged exposure may also damage food surfaces and degrade bioactive compounds, negatively impacting color and nutrient content [8].

To overcome these limitations, researchers have developed plasma-activated water (PAW) technology, which uses water as a medium for indirect cold plasma treatment of food items. PAW avoids the physical damage to the food surface associated with direct plasma exposure [9]. PAW is typically generated via two main methods: transporting reactive plasma species into water through discharge-induced convection at the air–water or underwater interface, or combining dielectric barrier discharge (DBD) with microbubbles to infuse reactive species into the solution, resulting in lowered pH. The acidification of PAW mainly results from the dissolution of plasma-generated nitrogen oxides (NO_x_) into water, forming nitrite (NO_2_^–^), nitrate (NO_3_^–^), and related acidic nitrogen species, which lower the pH. This acidic and oxidative environment, together with reactive oxygen and nitrogen species (RONS) in PAW, enhances antimicrobial activity. Under acidic conditions, interactions between hydrogen peroxide (H_2_O_2_) and NO_2_^–^ can generate highly reactive RNS that contribute to microbial inactivation [10]. Moreover, the low pH of PAW may suppress the activity of enzymes related to browning, such as polyphenol oxidase (PPO) and peroxidase (POD), thereby helping maintain postharvest quality in fresh produce [11]. RONS in PAW can penetrate microbial cells, causing damage to DNA and proteins and inducing oxidative stress. However, dense biofilms and high bacterial cell concentrations can hinder the diffusion of reactive species, thus reducing the bactericidal efficacy of PAW [12].

In recent years, preservation methods assisted by ultrasound (US) have gained popularity in food processing due to their effectiveness in microbial inactivation and browning control. For instance, combining US technology with weakly acidic electrolyzed water severely damages the cell membrane of *Pseudomonas* spp., resulting in increased leakage of nucleic acids and proteins [13]. In addition, the combined application of US and ε-polylysine markedly inhibited microbial growth in fresh-cut lettuce while reducing weight loss, color changes, and the activities of POD and PPO [14]. In spinach, US and chlorine dioxide combination treatment significantly reduced NO_3_^–^ levels and maintained key quality indicators better than either treatment alone [15]. US generates acoustic streaming and microstreaming, which disrupts the diffusion boundary layer at the interface between the solid and liquid phases, increasing convection and rates of mass transfer. This potentially enables RONS in PAW to reach the wrinkles, gill interspaces, and micropores on the shiitake mushroom surface more rapidly and penetrate more deeply [16]. Additionally, cavitation bubble collapse can locally generate microjets, shock waves, and high shear stresses; these mechanical effects disrupt microbial adhesion and biofilm structures on rough surfaces, thereby expectedly enhancing the transport and penetration of RONS into the biofilm matrix and facilitating microbial inactivation [17].

**Fig. 1:**
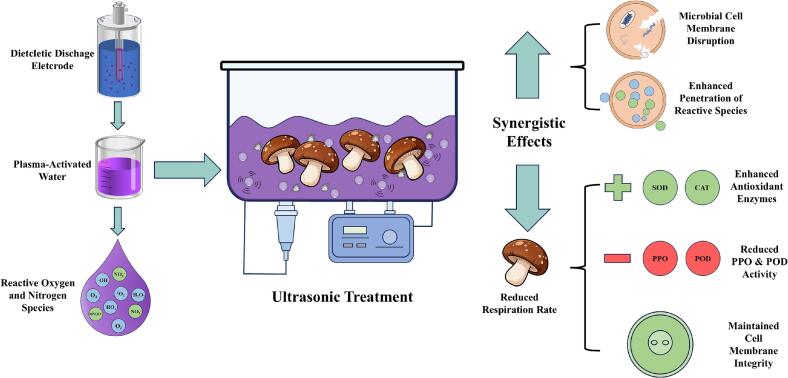
Graphical abstract.

Therefore, this study evaluates a hurdle technology combining PAW and US to explore their combined effects on shiitake mushroom preservation. Under refrigerated storage (4 °C for 14 d), this study systematically measured microbial load (aerobic plate count), macroscopic quality (firmness, weight loss, browning index, total soluble solids), physiological indicators (respiration rate, cell membrane permeability, malondialdehyde (MDA) content), and antioxidant and metabolic responses (total phenolic content, DPPH radical-scavenging activity, PPO and POD activities, superoxide dismutase (SOD) and catalase (CAT) activities). This comprehensive assessment aims to provide both a theoretical basis and practical processing parameters for applying PAW combined with US (PAW-US) treatment as an efficient non-thermal preservation technology to extend the postharvest shelf life of shiitake mushrooms Fig. 1.

## 2 Materials and methods

### 2.1 Sample preparation

Fresh shiitake mushrooms (*Lentinus edodes*) were harvested at Shandong Qihe Biological Technology Co., Ltd. After pre-cooled at 4 ℃ the samples were transported to our cold storage facility (4 ± 1 ℃) at Shandong University of Technology via cold chain. A batch of shiitake mushrooms were selected, ensuring that they were not rotten, damaged, or diseased and with uniformity in maturity, appearance, shape, and size.

### 2.2 Experiments design

#### 2.2.1 Generation of PAW and PAW treatment

**Fig. 2:**
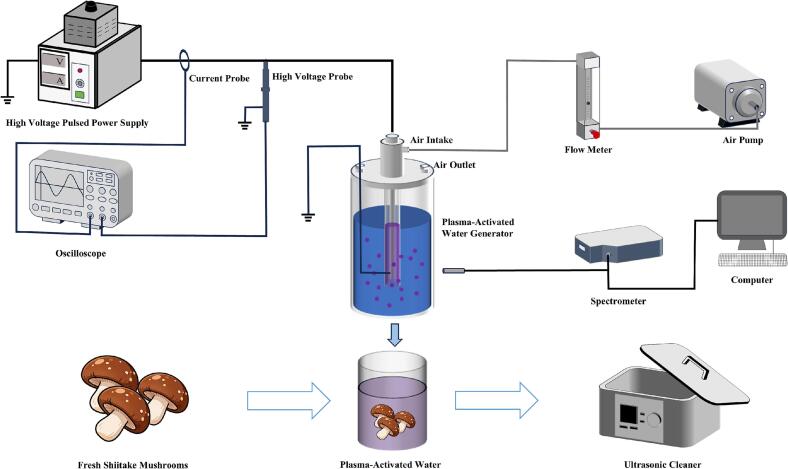
Schematic diagram of shiitake mushrooms processing via PAW-US treatment.

The method for PAW generation and processing was based on our previous work [18], with the specific operating parameters described below. PAW was generated using a DBD system consisting of a coaxial quartz-tube reactor, a high-voltage power supply (CTP-2000KP; Nanjing Suman Plasma Technology Co., Ltd., China), an air pump, and an optical and electrical diagnostic system (Fig. 2). The reactor comprised two coaxially aligned quartz tubes separated by an annular discharge gap, with a cylindrical stainless-steel high-voltage electrode embedded in the inner quartz tube (sealed at the bottom) and a copper mesh grounded electrode wrapped around the outer quartz tube. The discharge zone length was 12 cm. Air at atmospheric pressure was used as the working gas at 4 L min^−1^ and introduced into 2 L of deionized water contained in a quartz vessel in the form of plasma bubbles for 25 min. The optical and electrical characteristics of the discharge were analyzed using an optical fiber spectrometer (BRC115P-U, B&W Tek, Newark, DE, USA), a high-voltage probe, a current probe, and an oscilloscope (TBS1107; Tektronix, Inc., USA). Discharge power was calculated from the Lissajous figure using a 0.47 µF capacitor connected in series at the power supply output. The applied alternating-current voltage had a peak value of 13.9 kV and a frequency of 15.6 kHz, and the discharge power was 126 W. PAW generation was conducted at room temperature, and the solution temperature was monitored in real time using a thermometer without active temperature control.

US treatment was performed using an ultrasonic bath operating at a frequency of 40 kHz in continuous mode (duty cycle: 100 %). Three actual acoustic power levels were applied, namely 110, 220, and 330 W. Because the ultrasonic bath was equipped with a function for temperature monitoring, the treatment temperature was monitored in real time during sonication, and a water cooling circulation system was used to minimize temperature rise during treatment.

The fresh shiitake mushrooms were randomly assigned to 7 groups (n = 280 /group). The fresh shiitake mushrooms were placed in a 1 L PAW glass container until they were completely submerged and then immediately treated under different US powers (i.e., 110, 220, and 330 W) for 10 min until all the mushrooms were processed, which were respectively labelled as PAW-US_110_, PAW-US_220_, and PAW-US_330_. The following 4 treatment types were administered: 1 L of PAW soaking treatment (PAW group), 1 L of distilled water soaking followed by immediate US treatment (US group), 1 L of distilled water-soaking treatment (Water group), and no treatment (Control group). The treatment duration for all sets was 10 min. After processing, the shiitake mushroom samples were dried using blotting paper and then air-dried naturally at 25 ℃ to remove excess moisture. All treatment groups were set up in triplicate. Subsequently, the samples were sealed with tray cling film and stored in complete darkness at 4 ± 1 °C under a 90 ± 5 % relative humidity for 14 d. Each group of samples was analyzed once every 2 d.

#### 2.2.2 Physicochemical characteristics of PAW

The pH value, conductivity and oxidation–reduction potential (ORP) were measured with calibrated pH meters (FE20; Mettler Toledo International Inc., Switzerland), conductivity meters and an ORP meter (DDS-307A, YHBJ-262; Shanghai Yidian Scientific Instrument Co., Ltd., China) respectively [19]. The concentrations of NO_3_^–^ and NO_2_^–^ in the PAW group was measured by spectrophotometry (UV-2600; Shimadzu International Trading (Shanghai) Co., Ltd., China) [20]. The concentrations of H_2_O_2_ and O_3_ were determined using the titanium oxysulfate assay and the indigo trisulfonate colorimetric assay, respectively [21].

#### 2.2.3 Microbiological analysis of shiitake mushrooms

A sterile homogenizer was used to mix 10 g of shiitake mushroom samples with sterile diluent in a sterile homogenization bag for 2 min and analyzed as described by Liu et al., and the result was expressed as log CFU g^−1^ [22].

#### 2.2.4 Browning index of shiitake mushrooms

The color difference meter (NR60CP; Guangdong 3nh Intelligent Technology Co., Ltd., China) was used to measure the color of the shiitake mushroom caps. The L*, a*, and b* values were recorded. Six shiitake mushrooms were randomly selected for each treatment group and subjected to three random surface measurement points. The browning index was calculated according to equations (1), (2) given below [23]:

(1) $$\text{Browning index}=100\times \frac{X-0.31}{0.172}$$

(2) $$X=\frac{a^{*}+1.75\times L^{*}}{5.645\times L^{*}+a^{*}-3.012\times b^{*}}$$

In the formula, L* represents the brightness value; a* represents the red-green color value; and b* represents the yellow-blue color value.

#### 2.2.5 Respiration rate of shiitake mushrooms

For this assay, 8 shiitake mushrooms were sealed in a 1.3 L glass container, and the CO_2_ concentration was measured after 6 h of storage at 4 °C using an O_2_/CO_2_ analyzer (SCY-2A; Shanghai Xinrui Instrument Co., Ltd., China). The results were expressed as the respiration rate in mg CO_2_ kg^-1^h^−1^ [24].

#### 2.2.6 Firmness of shiitake mushrooms

Shiitake cap firmness was quantified by using the TA-XT texture analyzer (Brookfield Engineering Laboratories, Inc., USA) with the P/36R probe. During the test, the shiitake mushroom caps were compressed to 40 % of their original height while keeping the contact area constant. The test parameters were set to the following: a pre-test speed of 2 mm s^−1^, a test speed of 5 mm s^−1^, and a post-test speed of 5 mm s^−1^. Each stage lasted for 5 s, and the touch force was set at 5 g [25].

#### 2.2.7 wt loss of shiitake mushrooms

Shiitake mushrooms weight was quantified at 48 h intervals, and the weight loss was calculated using equation (3), as follows [26]:

(3) $$\text{Weight loss}\;\left(\%\right)=\frac{M_{0}-M_{1}}{M_{0}}\times 100\%$$

In the formula, *M*_0_ represents the initial weight of the sample, g; *M*_1_ represents the mass on each sampling day, g.

#### 2.2.8 Total soluble solids of shiitake mushrooms

Total Soluble solid content of shiitake mushrooms was quantified by using a handheld refractometer (HT111ATC; Shenzhen Yuanhengtong Technology Co., Ltd., China) [18].

#### 2.2.9 SOD and CAT activities of shiitake mushrooms of shiitake mushrooms

The activity of SOD was determined using the nitroblue tetrazolium reduction method described previously [27]. For the CAT activity, 1 g of shiitake mushrooms was added to 5 mL of sodium phosphate buffer (pH 7.5) and subjected to ice bath grinding first and then centrifugation at 4 ℃ for 20 min. After which, 100 µL of the supernatant was mixed with 2.9 mL of H_2_O_2_ solution. The change in the absorbance was recorded at 240 nm. Enzyme activity was expressed as units per gram fresh weight (U g^−1^) [28].

#### 2.2.10 Cell membrane permeability of shiitake mushrooms

Shiitake mushroom caps were sectioned into 2 mm slices, and aliquots of 10 g were immersed in distilled water for 30 min. A conductivity meter was used to measure the initial conductivity (*P*_0_) of this mixed sample. Next, the sample was allowed to stand at room temperature for 10 min, and the conductivity (*P*_1_) was measured again. Finally, the sample was boiled for 30 min and its final conductivity (*P*_2_) was determined [29]. Finally, the cell membrane permeability was calculated as per equation (4) given below:

(4) $$\text{Cell membrane permeability (\textbackslash{}\%)}\;=\frac{P_{1}-P_{0}}{P_{2}-P_{0}}\times 100\%$$

In the formula, *P*_0_ represents the conductivity of the mixed sample (measured in μS cm^−1^); *P*_1_ represents the conductivity of the sample at room temperature (measured in μS cm^−1^); *P*_2_ represents the conductivity of the sample after boiling (measured in μS cm^−1^).

#### 2.2.11 MDA content of shiitake mushrooms

For this assay, 1 g of the shiitake sample was homogenized with 5 mL of 10 % (w/v) trichloroacetic acid solution at 4 ℃, followed by centrifugation at 10,000 × g for 20 min using a centrifuge (TGL20MW; Hunan Herexi Instrument & Equipment Co., Ltd., China). Then, 2 mL of the supernatant was mixed with 2 mL of 0.67 % thiobarbituric solution, vortexed, and heated at 100 °C for 20 min. After cooling to an ambient temperature and centrifugation, the absorbance of the resulting supernatant was measured at 450, 532, and 600 nm, and MDA content was expressed as µmol kg^−1^ [30].

#### 2.2.12 PPO and POD activities of shiitake mushrooms

Briefly, 1 g of shiitake mushrooms and 5 mL of acetic acid-sodium acetate buffer solution (pH 5.5) were added and the resulting solution was ground under ice bath. The mixture was then centrifuged at 4 ℃ for 20 min, and the supernatant was added to the substrate to initiate the reaction. The activities of PPO and POD were determined by measuring the changes in their respective absorbance at 420 nm and 470 nm within 3 min. One active unit (1 U) of the enzyme activity was defined as the amount of enzyme required per gram of fresh shiitake mushroom to cause an increase of 0.01 in the absorbance value per minute, and was expressed as U g^−1^ [31], [32].

#### 2.2.13 Total phenolic content of shiitake mushrooms

For this assay, 1 g of shiitake mushroom caps was homogenized with 4 mL of 80 % ethanol, followed by centrifugation of the homogenate at 10,000 × g for 20 min. Then, 1 mL of the supernatant was mixed with 4 mL of the diluted 10-fold Folin-Ciocalteu reagent thoroughly for 3 min, after which the mixture was treated with 5 mL of 7.5 % Na_2_CO_3_ solution and placed in the dark for 1 h. Finally, the absorbance was read at 760 nm and quantified with reference to the gallic acid standard curve. The total phenol content was expressed as gallic acid equivalent per kilogram of the fresh sample (g GAE kg^−1^) [33].

#### 2.2.14 DPPH radical-scavenging activity of shiitake mushrooms

For this assay, 0.2 g of the shiitake mushroom samples was treated with 1 mL of 80 % ethanol solution and homogenized in an automatic grinder and centrifuged at 4 ℃. Then, 0.8 mL of the supernatant was mixed with 3.2 mL of the 0.1 mM DPPH solution, and the mixture was incubated in the dark for 30 min, followed by measurement of the absorbance at 517 nm with a spectrophotometer, using 80 % ethanol as the blank [34]. The DPPH radical-scavenging activity was calculated using Equation (5) given below:

(5) $$\text{DPPH radical scavenging activity}\;\left(\%\right)=1-\frac{\mathrm{Ab}s_{\text{sample}}}{\mathrm{Ab}s_{\text{control}}}\times 100\%$$

In the formula, *Abs*_control_ represents the absorbance of the control solution at 517 nm; *Abs*_sample_ represents the absorbance of the sample reaction mixture at 517 nm.

#### 2.2.15 Data processing and statistical analysis

A completely randomized design was applied to the present experiment. The data were expressed as the mean ± SD (n = 3). Statistical analyses were performed using SPSS 27.0, including Duncan's multiple-range test (*p* < 0.05) and Pearson’s correlation analysis.

## 3 Results and Discussion

### 3.1 Preparation and physicochemical characteristics of PAW

**Fig. 3:**
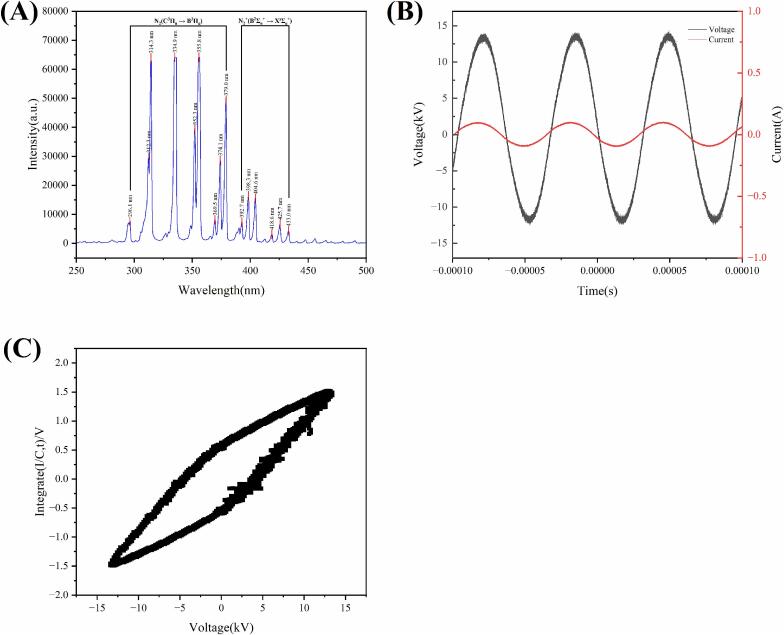
(A) Optical emission characteristics of air plasma; (B) discharge voltage and current waveform diagram. (C) Lissajous figure.

As shown in Fig. 3A. The plasma emission spectrum is primarily composed of intense emission peaks from the second positive system (SPS; C^3^Π_u_ → B^3^Π_g_) of nitrogen molecules, accompanied by weaker peaks from the first negative system (FNS; B^2^Σ_u_^+^→X^2^Σ_g_^+^) of nitrogen molecular ions. The instantaneous discharge voltage and current waveforms are presented in Fig. 3B, and the corresponding Lissajous figure is shown in Fig. 3C. Under the present operating conditions, the discharge power was calculated to be 126 W.

After 5 min of treatment, the pH of PAW dropped to 4.33, while that of distilled water remained neutral (Table 1). Conductivity also increased substantially with extended activation time, although the rate of increase slowed after 25 min of plasma treatment (Table 1). With the extension of activation time, the ORP of PAW demonstrates a continuous upward trend, reaching 338.5 mV after 30 min of activation (Table 1). The antibacterial activity of PAW is primarily attributed to charged ions, ROS, RNS, and free radicals. The production of these reactive substances contributes to the observed increase in conductivity and ORP [35]. After 5 min of treatment, the NO_3_^–^ concentration increased from 6.41 mg L^−1^ to 34.36 mg L^−1^ with longer treatment duration (Table 1). The increase was gradual during the first 15 min and accelerated sharply from 15 to 25 min, after which it plateaued. NO_2_^–^ levels rose rapidly to 3.3 mg L^−1^ in the first 5 min and remained relatively stable thereafter (Table 1).

**Table 1:** Physiochemical properties of plasma activated water.

Activation time (min)	pH	Electrical conductivity (µS cm^−1^)	ORP (mV)	NO_3_^–^ concentration (mg L^−1^)	NO_2_^–^ concentration (mg L^−1^)	H_2_O_2_ concentration (mg L^−1^)	O_3_ concentration (mg L^−1^)
0	7.06 ± 0.05 a	0.46 ± 0.06 g	90.43 ± 3.75f	0 ± 0 g	0 ± 0c	0 ± 0 g	0 ± 0f
5	4.33 ± 0.05b	7.27 ± 0.48f	190.03 ± 5.68 e	6.41 ± 0.34f	3.3 ± 0.18b	4.97 ± 1.00f	0.07 ± 0.02 e
10	3.87 ± 0.03c	16.59 ± 1.18 e	259.07 ± 4.52 d	7.20 ± 0.44 e	3.55 ± 0.14 ab	15.93 ± 1.17 e	0.15 ± 0.05 d
15	3.70 ± 0.04 d	23.27 ± 1.15 d	301.67 ± 3.98c	9.14 ± 0.50 d	3.53 ± 0.16 ab	23.33 ± 1.10 d	0.4 ± 0.04c
20	3.59 ± 0.04 e	35.42 ± 0.75c	320.8 ± 3.76b	14.61 ± 0.29c	3.56 ± 0.20 ab	34.67 ± 0.80c	0.49 ± 0.04b
25	3.42 ± 0.03f	46.77 ± 1.06b	331 ± 4.42 a	27.64 ± 0.47b	3.63 ± 0.17 a	38.53 ± 1.31b	0.6 ± 0.04 a
30	3.36 ± 0.03f	51.40 ± 0.91 a	338.5 ± 4.30 a	34.36 ± 0.68 a	3.67 ± 0.15 a	41.57 ± 1.04 a	0.65 ± 0.05 a

Results are expressed as mean ± standard deviation. Vertical bars represent the standard deviation of the means of three replicates. Different lower-case letters indicate the statistically significant difference in the same column (*p* < 0.05).

H_2_O_2_ and O_3_ are two key long-lived reactive species in PAW. Among them, H_2_O_2_ is a relatively stable reactive species in PAW, and its concentration generally increases with prolonged plasma treatment time while remaining relatively stable during an extended storage period after treatment [10]. Under the present treatment conditions (H_2_O_2_ = 38.53 mg L^−1^, contact time = 10 min, PAW volume-to-mass ratio = 4 mL g^−1^), H_2_O_2_ was considered an important contributor to the oxidative capacity of PAW and may contribute to antimicrobial activity, whereas excessive H_2_O_2_ exposure may induce oxidative stress, leading to lipid peroxidation, protein damage, and loss of membrane integrity. At lower levels, H_2_O_2_ may additionally function as a signaling molecule in biological tissues [36]. As shown in Table 1, the H_2_O_2_ concentration increased with activation time, reaching 34.67 mg L^−1^ after 20 min, after which the increase became less pronounced, possibly due to a balance between H_2_O_2_ formation and its concurrent decomposition and consumption during activation. Given that H_2_O_2_ exposure can induce tissue injury in a dose-dependent manner and that US may enhance PAW antimicrobial efficacy by improving access and penetration of reactive species, the present PAW-US treatment should therefore be interpreted as a balance related to dose between microbial inactivation and potential tissue oxidative injury and quality loss [17], [37]. The concentration of O_3_ also shows a similar trend, increasing rapidly within the first 15 min before its generation rate gradually slows down (Table 1). This may be due to a series of reactions between H_2_O_2_, NO_2_^–^, and O_3_ in the PAW system, as well as the intensified reactions and accelerated consumption and volatilization of O_3_ resulting from the rising temperature [38]. These results suggest that the physicochemical properties of PAW are influenced by plasma activation time.

### 3.2 Aerobic plate count of shiitake mushrooms

**Fig. 4:**
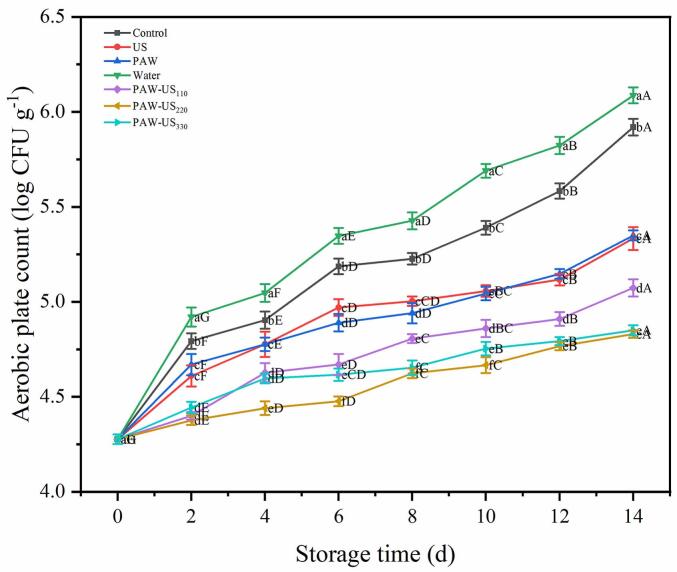
Effect of different treatment methods on the aerobic plate count of shiitake mushrooms during Storage. Average values (n = 3) with standard deviation are provided.

The natural microbiota on Shiitake mushrooms (*Lentinus edodes*) contributes to postharvest browning and tissue softening. Bacterial metabolites disrupt cellular membrane integrity, facilitating the interaction between tyrosinase and endogenous phenolic compounds. This enzymatic oxidation leads to the formation of quinones, which subsequently polymerize into melanin-like pigments, manifesting as visible browning symptoms. During the 14 d of storage, aerobic plate count levels increased in all groups. The log CFU g^−1^ increases for the Control, US, PAW, Water, PAW-US_110_, PAW-US_220_, and PAW-US_330_ groups were 1.64, 1.05, 1.07, 1.81, 0.79, 0.55, and 0.57, respectively. Notably, compared to the Control group, the PAW-US treatment could markedly delay the growth of bacteria (Fig. 4).

This reduction may be associated with the combined action of RONS in PAW and the physical effects related to US. US disrupt microbial surface structures and increase membrane permeability, thereby facilitating the entry of reactive species into microbial cells. In addition, effects related to cavitation are likely to enhance the availability of residual reactive species in PAW, further promoting intracellular RONS accumulation [39]. Excessive RONS subsequently induces oxidative stress, disrupts cellular metabolism, and ultimately leads to microbial inactivation [40]. Therefore, the lower aerobic plate count observed under PAW-US treatment is related to the above processes.

### 3.3 Browning index of shiitake mushrooms

The browning of shiitake mushrooms is primarily catalyzed by PPO, which oxidizes phenolic compounds to quinones. Additionally, natural microbial communities cause browning and tissue softening in these mushrooms. *Pseudomonas* spp. play a pivotal role in abnormal pigment deposition on mushrooms. They secrete specific toxins to destroy the cell membrane structure, causing tyrosinase to leak from vacuoles and interact with phenolic substances. This triggers oxidative reactions, wherein quinone compounds are formed. Ultimately, tissue browning occurs through the melanin-like formation pathway [41].

**Fig. 5:**
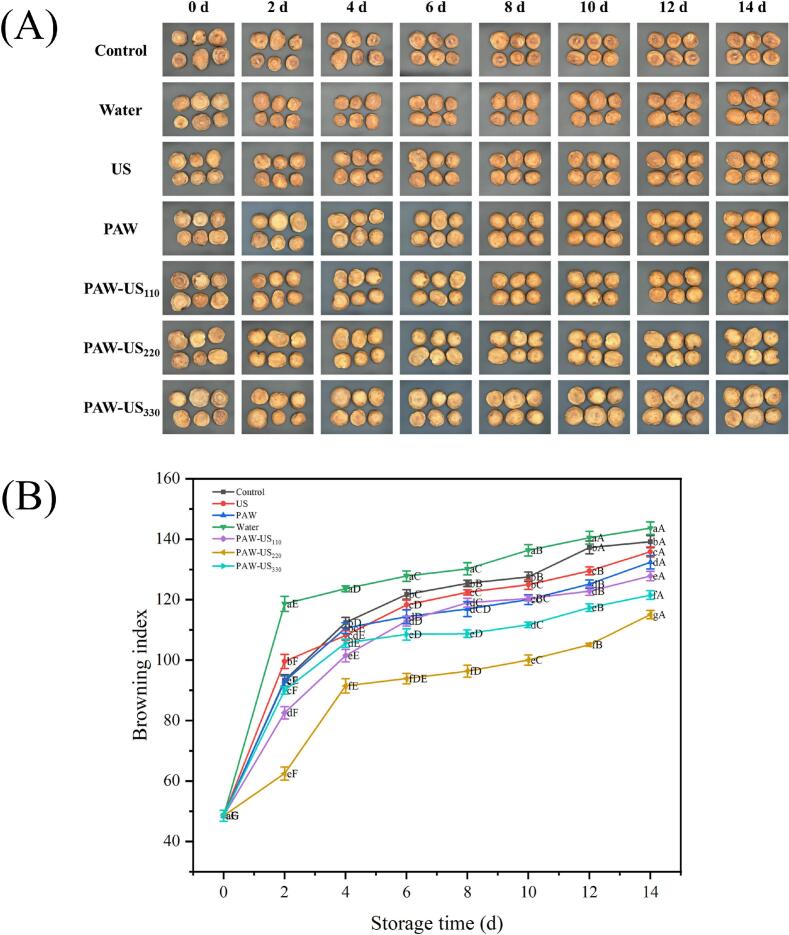
Morphological and visual changes in shiitake mushrooms under different treatment methods over a 14 d period. Average values (n = 3) with standard deviation are provided.

During the initial 4 d of storage, the browning index in all treatment groups increased rapidly, followed by a slower rate of increase in the later stages (Fig. 5A and 5B). This trend reflects the relatively high availability of browning substrates at the beginning of storage. As cells gradually senesced and their compartmentalization was disrupted, enzymes involved in browning and their substrates could come into contact more readily, thereby accelerating browning development [42]. After 4 d, the slower increase in browning is partly explained by reduced substrate availability. In addition, the accumulation of browning products, such as quinones, further influences browning progression through subsequent non-enzymatic reactions. Collectively, these factors contribute to the gradual slowdown of browning development [43]. At day of 14, all treatment groups exhibited browning to varying degrees. The Water group had the highest browning index (143.65), followed by the Control group (139.17). By contrast, the PAW-US groups showed lower browning indices, with reductions of 8 %, 17 %, and 13 %, respectively, compared to the Control group.

This inhibitory effect may be attributed to at least two factors. First, PAW-US treatment reduced PPO and POD activities, which are closely associated with browning development. Second, physical effects related to US, including cavitation, may facilitate the access of reactive species in PAW to microbial cells by disrupting microbial surface structures or increasing membrane permeability, thereby inhibiting microbial proliferation and contributing to reduced enzymatic browning [44].

### 3.4 Respiration rate of shiitake mushrooms

**Fig. 6:**
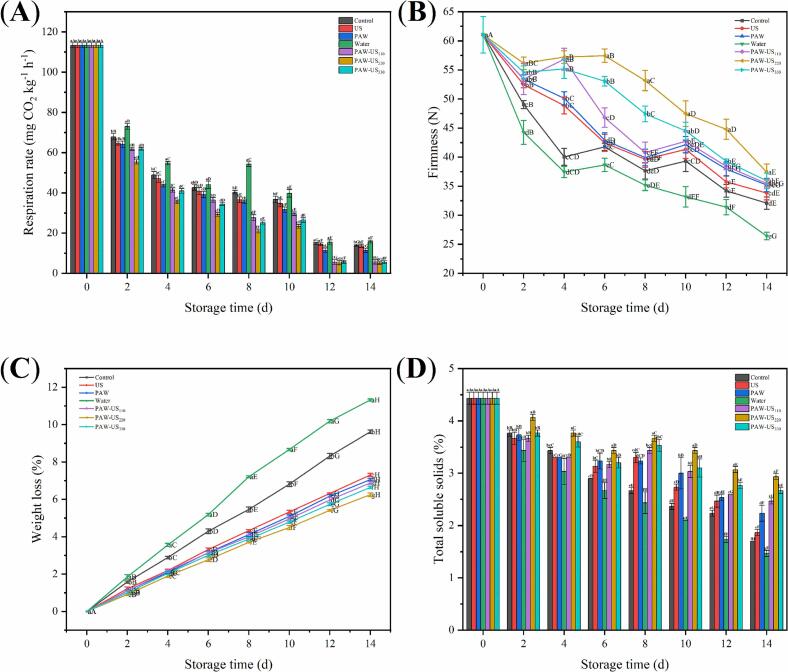
Effect of different treatment methods on the (A) respiration rate, (B) firmness, (C) weight loss, and (D) total soluble solids of shiitake mushrooms during Storage. Average values (n = 3) with standard deviation are provided.

Respiration in fresh shiitake mushrooms consumes internal nutrients to maintain metabolic processes. A higher respiration rate leads to faster nutrient depletion, tissue senescence, and decreased storage stability. Over the storage period, respiration rates declined as internal reserves in the mushrooms were exhausted (Fig. 6A). On day 8, respiration rates for the Control, Water, US, PAW, PAW-US_110_, PAW-US_220_, and PAW-US_330_ groups were 40.31, 54.28, 36.55, 35.53, 27.65, 20.99, and 25.24 mg CO_2_ kg^-1^h^−1^, respectively. Notably, the PAW-US groups exhibited lower respiration rates than the Control and Water groups. The PAW-US_220_ group maintained a relatively low respiration rate throughout the storage period. This reduction may be associated with enhanced mass transfer of RONS in PAW under US treatment, which improves their interaction with shiitake mushroom tissues and contributes to better maintenance of tissue integrity and reduced physiological deterioration, thereby leading to lower respiration during storage [45]. After 12 d, respiration rates in all groups began to stabilize, likely due to the increased mushroom spoilage.

### 3.5 Firmness, weight loss and total soluble solids of shiitake mushrooms

Postharvest softening of shiitake mushrooms is primarily caused by microbial degradation, which break down the intracellular matrix and compromise the integrity of central vacuoles [46]. Throughout the storage period, shiitake mushrooms treated with PAW-US treatment consistently retained higher firmness than both the Water and Control groups (Fig. 6B). On day 8, firmness in the PAW-US_110_, PAW-US_220_, and PAW-US_330_ groups was higher by 10.5 %, 16.6 %, and 11.9 %, respectively than that of the Control group. By day 14, firmness in the Control and Water groups had decreased by 47 % and 57 %, respectively, from their initial values. By contrast, PAW-US treatment effectively slowed the softening process, with the PAW-US_220_ group maintaining firmness 17 % higher than the Control group and 42 % higher than the Water group. The maintenance of firmness in shiitake mushrooms is associated with the combined effects of PAW and US. RONS in PAW suppress microbial proliferation, thereby reducing microbially induced tissue deterioration. Meanwhile, US treatment may enhance antioxidant defense and contribute to the maintenance of cell wall integrity, which may partially explain the delayed tissue softening observed during storage [47].

As shown in Fig. 6C, weight loss increased continuously in all treatment groups during storage. This trend is associated with ongoing transpiration and respiratory metabolism in shiitake mushrooms, which promote moisture loss from the tissue to the surrounding environment. Additionally, microbial degradation of shiitake mushroom tissue structure reduces its water-holding capacity, thereby promoting moisture loss and accelerating weight loss [48]. By day 14, weight loss for the Control, US, PAW, Water, PAW-US_110_, PAW-US_220_, and PAW-US_330_ groups were 9.62 %, 7.31 %, 7.06 %, 11.32 %, 6.88 %, 6.25 %, and 6.64 %, respectively. PAW-US treatment reduced weight loss in shiitake mushrooms compared with both the Control and Water groups. This effect was associated with lower respiration, reduced microbial deterioration, and a decreased rate of moisture loss during storage [49]. Among the combined treatments, PAW-US_220_ exhibited the lowest weight loss compared with both the PAW-US_110_ and PAW-US_330_ groups. These results suggest that an appropriate US power level improves the preservation performance of PAW-US treatment in shiitake mushrooms. This effect is related to a greater suppression of respiratory metabolism and surface microbial growth, thereby reducing weight loss during storage.

The total soluble solids content directly reflects the quality of shiitake mushrooms, typically decreasing during storage due to sugar consumption via respiration. As shown in Fig. 6D, during the first 6 d, all treatment groups exhibited a sharp decline in total soluble solids. However, from days 6 to 8, all groups except the Control and Water groups showed an increase in total soluble solids, which is related to reduced microbial consumption of nutrients under the treatments and the release of soluble components associated with tissue softening or structural changes [50]. By the end of storage, PAW-US treatment improved total soluble solids content in shiitake mushrooms. The PAW-US_220_ group had the highest total soluble solids content, measuring 99 % higher than the Water group and approximately 72 % higher than the Control group. This effect is related to the lower respiration rate and the reduced consumption of sugars and organic acids under PAW-US treatment [51].

### 3.6 SOD and CAT activities of shiitake mushrooms

**Fig. 7:**
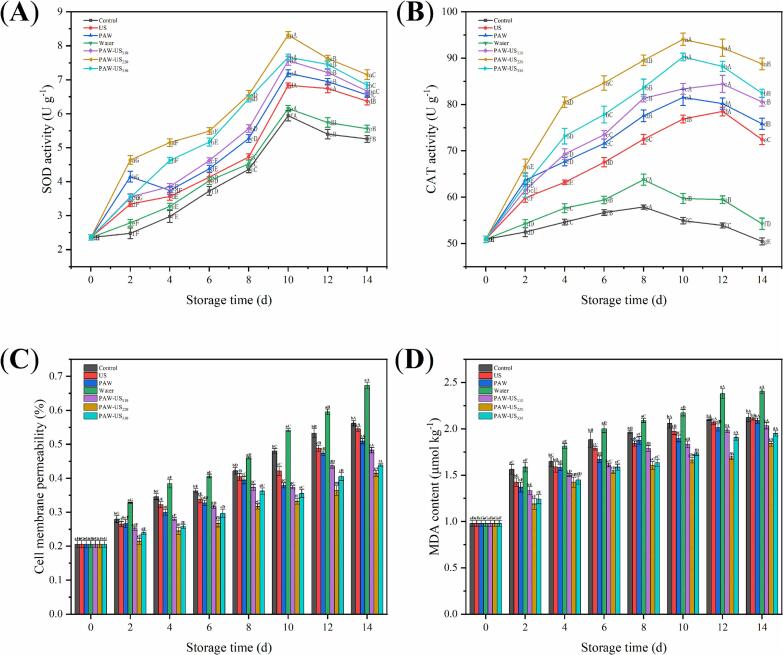
Effect of different treatment methods on the (A) SOD activity, (B) CAT activity, (C) cell membrane permeability, (D) MDA content of shiitake mushrooms during Storage. Average values (n = 3) with standard deviation are provided.

SOD serves as a primary defense mechanism in the antioxidant system, functioning in conjunction with CAT to maintain intracellular redox balance and alleviate oxidative stress [52]. During the early storage period, shiitake mushroom cells regulate SOD activity to combat this stress (Fig. 7A). By day 8 of storage, SOD activity in the PAW-US_110_, PAW-US_220_, and PAW-US_330_ groups was 21.4 %, 28.6 %, and 22.5 % higher, respectively, than in the untreated Control group. However, as storage progressed, SOD activity gradually declined, which may weaken the capacity to scavenge ROS and thereby contribute to increased oxidative stress and oxidative damage. Despite this decline, by day 14, the PAW-US_110_, PAW-US_220_, and PAW-US_330_ groups still maintained higher SOD activity levels than the Control group, with respective increases of 21 %, 26.4 %, and 23.1 %. Notably, the PAW-US_220_ group exhibited the highest SOD activity throughout the storage period.

CAT activity in all treatment groups followed an increasing-then-decreasing trend during storage (Fig. 7B). On day14, the PAW-US_220_ and PAW-US_330_ groups exhibited higher CAT activity than the Control group, with increases of 43 % and 39 %, respectively. Elevated CAT activity in shiitake mushroom tissue contributes to the reduction of cellular damage induced by oxidative stress, which could support the maintenance of tissue integrity and overall quality during storage [53].

These results indicate that the PAW-US treatment increased antioxidant enzyme activities in shiitake mushrooms during storage. It is hypothesized that US might enhance the penetration of ROS in PAW, potentially allowing them to access membrane channels and induce endogenous ROS generation, which could lead to intracellular ROS accumulation [17]. As a potential adaptive response to prevent oxidative damage to cellular structures and biomacromolecules, the shiitake mushrooms upregulate the levels of antioxidant enzymes (SOD and CAT) as well as nonenzymatic antioxidant compounds to resist oxidative stress and scavenge free radicals [54].

### 3.7 Cell membrane permeability and MDA content of shiitake mushrooms

Environmental stress or mechanical damage can compromise the structural integrity of plant cell membranes, leading to disrupted osmotic regulation, electrolyte leakage, and decreased membrane fluidity. These changes can be effectively quantified through conductivity measurements [55]. As shown in Fig. 7C, cell membrane permeability increased in all groups during storage. However, the rise was lower in the PAW-US treatments compared to the Control and Water groups. On day 14, the permeability rates for the Control, Water, US, PAW, PAW-US_110_, PAW-US_220_, and PAW-US_330_ groups were 56 %, 67 %, 55 %, 51 %, 48 %, 41 %, and 44 %, respectively. Among these treatments, PAW-US_220_ showed the strongest protective effect, suggesting that an appropriate PAW-US treatment maintain membrane integrity and reduce cellular damage during storage.

MDA serves as a marker of oxidative damage, as it can bind to proteins and nucleic acids, causing their inactivation. Excessive MDA accumulation can disrupt membrane permeability and damage organelle structures [56]. During storage, MDA content in shiitake mushrooms steadily increased, showing a trend consistent with that of cell membrane permeability (Fig. 7D). This sustained upward trajectory indicates progressive deterioration of cell membrane integrity. Compared to the initial values, MDA levels increased by 59 % in the Water group and 54 % in the Control group. By contrast, the increase was less pronounced in the PAW-US groups, ranging from 46.7 % to 51.7 %. Throughout the storage period, MDA content in all PAW-US groups remained consistently lower than that in the Control group. On day 14, the PAW-US_220_ group exhibited 24 % and 13 % lower MDA content than the Water and Control groups, respectively, suggesting that PAW-US treatment has alleviated membrane lipid peroxidation and reduced oxidative damage in shiitake mushrooms during storage.

In conclusion, the lower membrane permeability and MDA content observed in the PAW-US groups suggest that the combined treatment mitigates membrane deterioration and oxidative damage during storage. This protective effect, associated with the combined action of reactive species in PAW and the physical effects related to US, may enhance antioxidant defense and help maintain the balance between free radical generation and scavenging in shiitake mushrooms [57], [58]. Consequently, PAW-US treatment delays postharvest senescence and browning, thereby contributing to improved storage quality and shelf life.

### 3.8 PPO and POD activities of shiitake mushrooms

**Fig. 8:**
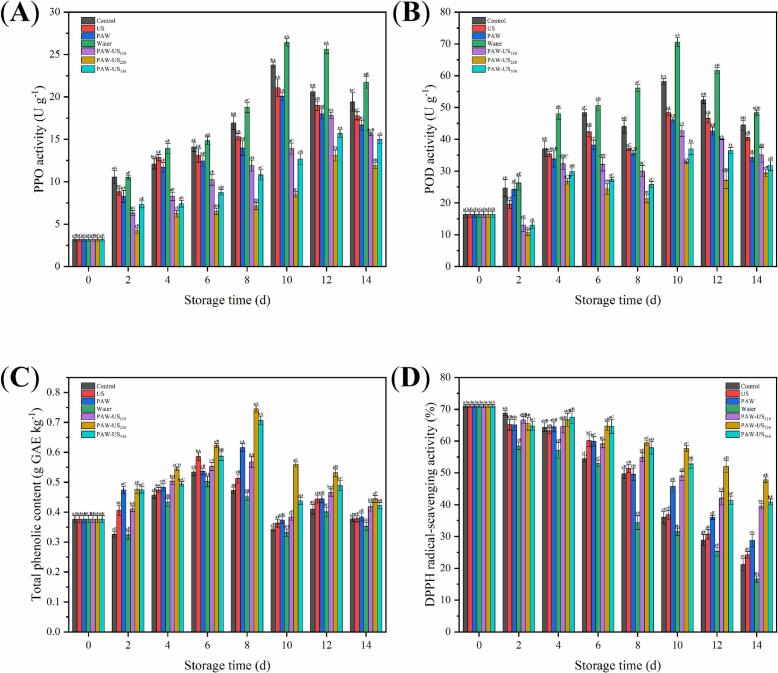
Effect of different treatment methods on the (A) PPO activity, (B) POD activity, (C) total phenolic content, (D) DPPH radical scavenging activity of shiitake mushrooms during Storage. Average values (n = 3) with standard deviation are provided.

PPO catalyzes the oxidation of phenolic compounds to quinones, which polymerize to form melanin, leading to browning and quality degradation in shiitake mushrooms. As freshness declines during storage, PPO activity typically increases, intensifying browning. However, during advanced spoilage, PPO activity may decline (Fig. 8A). During storage, in the Control, Water, US, and PAW groups, PPO activity peaked on day 10 of storage, whereas in the PAW-US groups (PAW-US_110_, PAW-US_220_, PAW-US_330_), peak activity occurred on day 12. Among all groups, the Water group showed the highest PPO activity, followed by the Control group. By contrast, PAW-US treatments, particularly PAW-US_220_, suppressed PPO activity throughout storage. On day 10, PPO activity in the PAW-US_110_, PAW-US_220_, and PAW-US_330_ treatment groups was 42 %, 64 %, and 47 %, respectively, lower than in the Control group. By day 14, the reductions were 19 %, 39 %, and 23 %, respectively.

The shiitake mushrooms continue their metabolic activities after harvest. POD is a defense-related enzyme whose activity increases in response to cellular stress and damage. POD plays a critical role in scavenging ROS and reducing oxidative damage, thus preserving cellular stability. As storage time increases, the mushrooms undergo deterioration, causing a decline in POD activity (Fig. 8B). The Control and Water groups showed a faster rate in the early stage. On day 10, POD activities in the Control, Water, US, PAW, PAW-US_110_, PAW-US_220_, and PAW-US_330_ groups were 58.2, 70.6, 48.4, 46.2, 42.6, 32.7, and 37.0 U g^−1^, respectively. PAW-US groups (PAW-US_110_, PAW-US_220_, and PAW-US_330_) exhibited lower POD activity, with PAW-US_220_ showing the greatest suppression. After storage for 10 d, although POD activity declined in all groups, the PAW-US treatments continued to maintain lower activity than the Control and Water groups.

PAW-US treatment effectively inhibits PPO and POD activities in shiitake mushrooms during storage. We hypothesize that this suppression might be associated with potential conformational changes or inactivation of both PPO and POD enzymes, likely induced by ROS in PAW and the cavitation effects of US [11], [59].

### 3.9 Total phenolic content of shiitake mushrooms

Total phenolic content is a key indicator of the nutritional and functional properties of shiitake mushrooms. By day 8, total phenolic content had increased in all treatment groups relative to day 0, and the increase was greater in the PAW-US treatments than in PAW or US alone (Fig. 8C). The Water group exhibited the lowest phenolic content, followed by the Control group. The PAW-US_220_ group reached the highest phenolic level, with increases of 58 % and 65 % compared with the Control and Water groups, respectively. This enhancement is related to the induction of phenolic biosynthesis, potentially involving the phenylpropanoid pathway, thereby contributing to higher phenolic accumulation [60]. By contrast, phenolic content in the Water and Control groups declined after day 8, likely due to phenolics participating in enzymatic browning. All treatment groups showed a downward trend in phenolic content after day 8, possibly due to increased cell membrane permeability and lipid peroxidation. Nevertheless, by the end of storage, the PAW-US groups maintained an advantage, with the PAW-US_220_ group showing 18 % and 26 % higher total phenolic content than the Control and Water groups, respectively.

### 3.10 DPPH radical-scavenging activity of shiitake mushrooms

The DPPH radical scavenging activity is a critical indicator of antioxidant activity in shiitake mushrooms. Antioxidant activity is a critical determinant of preservation efficacy because oxidative reactions are primary contributors to postharvest deterioration, such as browning, nutrient loss, and flavor degradation. As shown in Fig. 8D, the DPPH radical scavenging activity in all treatment groups gradually declined over the storage period. The Water group consistently exhibited the lowest rate compared with the other groups. However, the PAW-US groups (PAW-US_110_, PAW-US_220_, and PAW-US_330_) effectively delayed this decline compared with the Control, Water, US, and PAW groups. By day 14 of storage, the PAW-US_110_, PAW-US_220_, and PAW-US_330_ groups showed DPPH radical scavenging activity 87 %, 126 %, and 93 % higher than the Control group, respectively. This enhanced antioxidant retention might be associated with higher antioxidant enzyme activities and increased total phenolic content, which may contribute to mitigating oxidative damage during storage and thereby support the maintenance of shiitake mushroom quality [61].

### 3.11 Correlation analysis

**Fig. 9:**
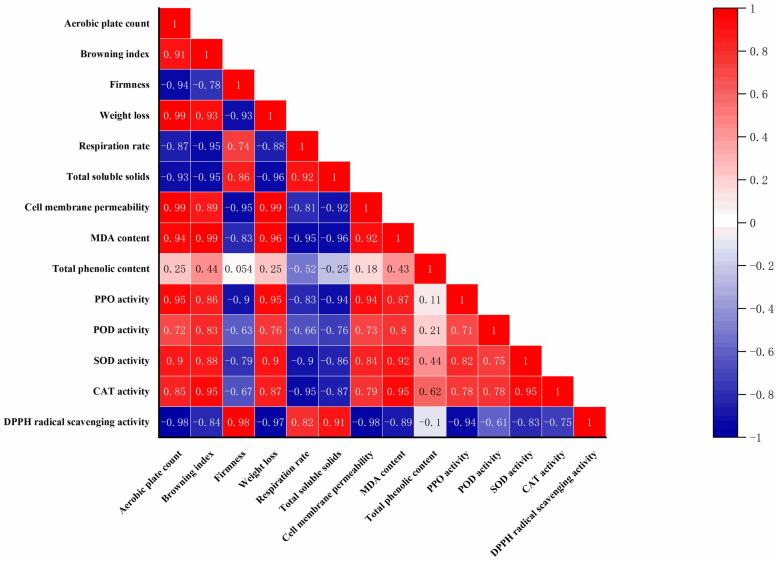
Correlation analysis of various indicators during the storage period of the PAW-US_220_ treatment group.

As shown in Fig. 9, the correlation heatmap was generated based on the temporal changes in indicators related to quality in the PAW-US_220_ group during the 14 d storage period, reflecting the dynamic associations among these indicators during storage. Overall, parameters related to deterioration, including aerobic plate count, browning index, weight loss, cell membrane permeability, and MDA content, showed strong positive correlations with each other. For example, aerobic plate count was highly positively correlated with weight loss (r = 0.99) and cell membrane permeability (r = 0.99), and the browning index was also strongly positively correlated with MDA content (r = 0.99), indicating that microbial proliferation, tissue water loss, membrane damage, and lipid peroxidation changed coordinately during storage under PAW-US_220_ treatment. Conversely, indicators related to quality retention, such as firmness, total soluble solids, and DPPH radical-scavenging activity, were generally negatively correlated with these parameters related to deterioration. For example, firmness was negatively correlated with cell membrane permeability (r = -0.95). In contrast, DPPH radical-scavenging activity showed strong negative correlations with aerobic plate count (r = -0.98) and cell membrane permeability (r = -0.98). This indicates that better texture retention and antioxidant capacity were associated with lower microbial load and reduced membrane damage during storage.

In addition, SOD and CAT activities were positively correlated with several parameters related to deterioration. For example, SOD activity was positively correlated with MDA content (r = 0.92), and CAT activity was also strongly positively correlated with MDA content (r = 0.95). This pattern suggests that an increase in antioxidant enzyme activities reflects a defensive response of shiitake mushroom tissue to oxidative stress during storage rather than an unequivocal improvement in quality. Overall, the correlation analysis indicates a close dynamic linkage among microbial changes, oxidative damage, membrane integrity, and quality deterioration in shiitake mushrooms stored under PAW-US_220_ treatment. These results further support the view that PAW-US treatment delays postharvest deterioration in shiitake mushrooms by suppressing microbial growth, alleviating oxidative damage, and maintaining structural integrity.

### 3.12 Effects of PAW composition and US treatment on postharvest quality

The preservation effects observed in this study may be associated with the combined action of PAW chemistry and US physical effects. Plasma activation created an acidic and oxidative environment, as reflected by the elevated ORP, which can be regarded as a macroscopic indicator of the overall oxidative capacity of the system. Under these conditions, US at 40 kHz and 220 W may act as a kinetic enhancer by reducing mass transfer resistance at the mushroom–liquid interface and facilitating the access of reactive species to the porous surface and internal microstructures of shiitake mushrooms.

This increased internal exposure may partly explain the lower PPO and POD activities, possibly through oxidative modification of enzyme active sites or related catalytic structures, thereby retarding the progression of enzymatic browning. Meanwhile, the coordinated changes in MDA content and SOD/CAT activities suggest that the treatment imposed a moderate and controllable level of oxidative stimulation rather than excessive oxidative injury, which may have helped maintain tissue integrity and texture during storage. Nevertheless, the specific mechanisms underlying PAW-US treatment, particularly the role of long-lived reactive species such as H_2_O_2_ in regulating postharvest physiological responses, still warrant further investigation.

## 4 Conclusion

This study investigated the effects of PAW combined with various US power levels (110, 220, and 330 W) on the postharvest quality of shiitake mushrooms. Among the tested treatments, PAW combined with 220 W US (PAW-US_220_) produced the most improvements. US treatment disrupts the membranes of pathogenic bacteria through cavitation, facilitating deeper cellular penetration of RONS from PAW. This combined action dramatically reduces the growth of spoilage and respiration rates, while increasing intracellular RONS concentrations, which further damages microbial membranes. Consequently, the combined treatment suppresses microbial proliferation and metabolic activity during storage. Additionally, the PAW-US_220_ treatment preserved mushroom firmness and moisture content, maintained tissue compactness and stability of cell membrane permeability, and retained higher levels of soluble solids, suggesting enhanced nutrient retention. PAW-US treatment might activate phenylpropanoid metabolism, thereby increasing total phenolic content, boosting antibacterial and antioxidant capacity. Furthermore, RONS in PAW stimulated antioxidant enzyme activity, increasing DPPH radical scavenging activity, while US-assisted cavitation disrupted PPO and POD activities, reducing oxidative damage and browning and softening during postharvest storage, thus improving the storage ability of mushrooms. This efficient non-thermal combined treatment offers a promising approach for extending the shelf life and maintaining the postharvest quality of shiitake mushrooms.

## CRediT authorship contribution statement

**Jun Shao:** Writing – review & editing, Writing – original draft, Supervision, Formal analysis, Data curation, Conceptualization. **Xue Huang:** Resources, Project administration. **Yanyin Guo:** Writing – review & editing, Supervision, Methodology, Conceptualization. **Lu Sun:** Resources, Conceptualization. **LuJun Zhang:** Supervision, Project administration. **Jie Li:** Supervision, Conceptualization. **Xinhe Zhao:** Writing – review & editing, Supervision, Funding acquisition, Conceptualization. **Zitong Zhao:** Writing – review & editing, Methodology, Funding acquisition, Conceptualization.

## Declaration of competing interest

The authors declare that they have no known competing financial interests or personal relationships that could have appeared to influence the work reported in this paper.

## Data Availability

Data will be made available on request.

## References

[1] Xia R., Wang Y., Hou Z., Li Y., Wang Z., Zhu J., Ren H., Guo Y., Xin G. (2024). Umami loss mechanism upon shiitake mushrooms under cold storage: revisiting the role of energy metabolism via integrated physiological and transcriptomic analysis. Postharvest Biol. Technol..

[2] Pourbagher R., Abbaspour-Fard M.H., Sohbatzadeh F., Rohani A., Pourbagher M. (2023). Effect of plasma-activated water generated by surface DBD on inactivation of pathogens *Pseudomonas tolaasii* and *Lecanicillium fungicola* and enhancement of storage quality of button mushroom. J. Food Process Eng.

[3] Asdullah H.U., Chen F., Hassan M.A., Abbas A., Sajad S., Rafiq M., Raza M.A., Tahir A., Wang D., Chen Y. (2024). Recent advances and role of melatonin in post-harvest quality preservation of shiitake (*Lentinula edodes*). Front. Nutr..

[4] Zheng C., Li J., Liu H., Wang Y. (2023). Review of postharvest processing of edible wild-grown mushrooms. Food Res. Int..

[5] Subramaniam S., Jiao S., Zhang Z., Jing P. (2021). Impact of post-harvest processing or thermal dehydration on physiochemical, nutritional and sensory quality of shiitake mushrooms. Compr. Rev. Food Sci. Food Saf..

[6] Weltmann K., von Woedtke T. (2017). Plasma medicine-current state of research and medical application. Plasma Phys. Control. Fusion..

[7] B. Murtaza, L. Wang, X. Li, M.K. Saleemi, M.Y. Nawaz, M. Li, Y. Xu, Cold plasma: a success road to mycotoxins mitigation and food value edition, Food Chem. 445 (2024) 138378, https://doi.org/10.1016/j.foodchem.2024.138378. 10.1016/j.foodchem.2024.138378 38383214

[8] Gu Y., Shi W., Liu R., Xing Y., Yu X., Jiang H. (2021). Cold plasma enzyme inactivation on dielectric properties and freshness quality in bananas. Innov. Food Sci. Emerg. Technol..

[9] Jaiswal M., Srivastava B. (2025). Optimization of plasma-activated water generation, antimicrobial efficacy assessment, and sequential application with pulsed light for enhancing microbial safety of fresh-cut pineapple. Postharvest Biol. Technol..

[10] Zhao Y., Patange A., Sun D., Tiwari B. (2020). Plasma-activated water: physicochemical properties, microbial inactivation mechanisms, factors influencing antimicrobial effectiveness, and applications in the food industry. Compr. Rev. Food Sci. Food Saf..

[11] Wang J., Cui Y., Zhang M., Wang L., Aihaiti A., Maimaitiyiming R. (2024). Pulsed-control plasma-activated water: an emerging technology to assist ultrasound for fresh-cut produce washing. Ultrason. Sonochem..

[12] Tiwari B., Dinesh S., Prithiviraj V., Yang X., Roopesh M.S. (2025). Bacterial biofilm inactivation by plasma activated nanobubble water. J. Water Process Eng..

[13] F. Li, Q. Zhong, B. Kong, N. Pan, X. Xia, Y. Bao, Synergistic effect and disinfection mechanism of combined treatment with ultrasound and slightly acidic electrolyzed water and associated preservation of mirror carp (*Cyprinus carpio l.*) During refrigeration storage, Food Chem. 386 (2022) 132858, https://doi.org/10.1016/j.foodchem.2022.132858. 10.1016/j.foodchem.2022.132858 35367791

[14] Fan K., Zhang M., Bhandari B., Jiang F. (2019). A combination treatment of ultrasound and ε-polylysine to improve microorganisms and storage quality of fresh-cut lettuce. LWT.

[15] Y. Mu, Y. Feng, L. Wei, C. Li, G. Cai, T. Zhu, Combined effects of ultrasound and aqueous chlorine dioxide treatments on nitrate content during storage and postharvest storage quality of spinach (Spinacia oleracea *l.*), Food Chem. 333 (2020) 127500, https://doi.org/10.1016/j.foodchem.2020.127500. 10.1016/j.foodchem.2020.127500 32693317

[16] Fernandes F.A.N., Rodrigues S. (2023). Ultrasound applications in drying of fruits from a sustainable development goals perspective. Ultrason. Sonochem..

[17] Charoux C.M.G., Patange A.D., Hinds L.M., Simpson J.C., O'Donnell C.P., Tiwari B.K. (2020). Antimicrobial effects of airborne acoustic ultrasound and plasma activated water from cold and thermal plasma systems on biofilms. Sci. Rep..

[18] Zhao Z., Wang X., Ma T. (2021). Properties of plasma-activated water with different activation time and its effects on the quality of button mushrooms (Agaricus bisporus). LWT.

[19] Yan Y., Zhu X., Hao M., Ji X., Shi M., Niu B. (2024). Understanding the multi-scale structure, physicochemical and digestive properties of extruded yam starch with plasma-activated water. Int. J. Biol. Macromol..

[20] Tian Y., Ma R., Zhang Q., Feng H., Liang Y., Zhang J., Fang J. (2015). Assessment of the physicochemical properties and biological effects of water activated by non-thermal plasma above and beneath the water surface. Plasma Process. Polym..

[21] Hadinoto K., Rao N.R.H., Astorga J.B., Zhou R., Biazik J., Zhang T., Masood H., Cullen P.J., Prescott S., Henderson R.K., Trujillo F.J. (2023). Hybrid plasma discharges for energy-efficient production of plasma-activated water. Chem. Eng. J..

[22] Liu C., Chen C., Jiang A., Sun X., Guan Q., Hu W. (2020). Effects of plasma-activated water on microbial growth and storage quality of fresh-cut apple. Innov. Food Sci. Emerg. Technol..

[23] Zhang S., Fang X., Wu W., Tong C., Chen H., Yang H., Gao H. (2022). Effects of negative air ions treatment on the quality of fresh shiitake mushroom (*Lentinus edodes*) during storage. Food Chem..

[24] Hu T., Zheng S., Liu Q., Li M., Chen J., Zhang H., Lin M., Lin H., Chen Y. (2025). Melatonin treatment maintains the quality properties and storability of carambola fruit by modulating energy metabolism. Food Chem..

[25] Wang Y., Mo Y., Li D., Xiang C., Jiang Z., Wang J. (2019). The main factors inducing postharvest lignification in king oyster mushrooms (*Pleurotus eryngii*): wounding and ROS-mediated senescence. Food Chem..

[26] Zhang Y., Feng X., Shi D., Tian X., Huang W., Liu Y. (2025). Effects of chitosan‑calcium coating on the physiological characteristics and browning of *Stropharia rugosoannulata* during postharvest storage. Food Res. Int..

[27] Qu T., Li B., Huang X., Li X., Ding Y., Chen J., Tang X. (2020). Effect of peppermint oil on the storage quality of white button mushrooms (Agaricus bisporus). Food Bioprocess Technol..

[28] Lin Q., Lu Y., Zhang J., Liu W., Guan W., Wang Z. (2017). Effects of high CO_2_ in-package treatment on flavor, quality and antioxidant activity of button mushroom (Agaricus bisporus) during postharvest storage. Postharvest Biol. Technol..

[29] Moradi C., Hosseini E., Rousta E. (2025). Bitter almond gum-fish gelatin conjugate coatings extend the storage of button mushrooms. Postharvest Biol. Technol..

[30] Shao P., Wu W., Chen H., Sun P., Gao H. (2020). Bilayer edible films with tunable humidity regulating property for inhibiting browning of Agaricus bisporus. Food Res. Int..

[31] Guo Y., Chen X., Gong P., Guo J., Deng D., He G., Ji C., Wang R., Long H., Wang J., Yao W., Yang W., Chen F. (2022). Effect of shiitake mushrooms polysaccharide and chitosan coating on softening and browning of shiitake mushrooms (*Lentinus edodes*) during postharvest storage. Int. J. Biol. Macromol..

[32] Liu Q., Cui X., Song Z., Kong W., Kang Y., Kong W., Ng T.B. (2021). Coating shiitake mushrooms (*Lentinus edodes*) with a polysaccharide from *Oudemansiella radicata* improves product quality and flavor during postharvest storage. Food Chem..

[33] Li D., Qin X., Tian P., Wang J. (2016). Toughening and its association with the postharvest quality of king oyster mushroom (*Pleurotus eryngii*) stored at low temperature. Food Chem..

[34] Dong S., Guo J., Yu J., Bai J., Xu H., Li M. (2022). Effects of electron-beam generated x-ray irradiation on the postharvest storage quality of Agaricus bisporus. Innov. Food Sci. Emerg. Technol..

[35] Hou C., Lai Y., Hsiao C., Chen S., Liu C., Wu J., Lin C. (2021). Antibacterial activity and the physicochemical characteristics of plasma activated water on tomato surfaces. LWT.

[36] Thana P., Buppan P., Theepharaksapan S., Yooyongsatit S., Sonhom W., Pakdee K., Jinpol A., Surinsuk S., Thummaraksa N., Matra K. (2026). Influence of pin-to-plane discharge modes on the physicochemical properties of single-pass flow-through plasma-activated water and its efficacy in agricultural applications. IEEE Access.

[37] Eicher-Sodo M., Gordon R., Zheng Y. (2019). Characterizing the phytotoxic effects of hydrogen peroxide on common microgreen species and lettuce cultivars. HortTechnology.

[38] Guo D., Liu H., Zhang X., Ma X., Shi Y., Mao J., Zhao Z., Tu Z. (2025). Inactivation and inhibition of botrytis cinerea by plasma-activated water long-lived species. J. Phys. D Appl. Phys..

[39] Hu Z., Xu W., Sun Y., Xu H., Xu J., Huang L., Yao W., Yu Z., Xie Y. (2024). Ultrasound-assisted activation of PAW residual radicals in the concurrent elimination of ARB and ARGs: process efficiency, mechanism and implication. Chem. Eng. J..

[40] Xu Y., Tian Y., Ma R., Liu Q., Zhang J. (2016). Effect of plasma activated water on the postharvest quality of button mushrooms, Agaricus bisporus. Food Chem..

[41] Liu F., Xu Y., Zeng M., Zhang Y., Pan L., Wang J., Huang S. (2023). A novel physical hurdle technology by combining low voltage electrostatic field and modified atmosphere packaging for long-term stored button mushrooms (Agaricus bisporus). Innov. Food Sci. Emerg. Technol..

[42] Wang C., Meng L., Zhang G., Yang X., Pang B., Cheng J., He B., Sun F. (2024). Unraveling crop enzymatic browning through integrated omics. Front. Plant Sci..

[43] Tilley A., Mchenry M.P., Mchenry J.A., Solah V., Bayliss K. (2023). Enzymatic browning: the role of substrates in polyphenol oxidase mediated browning. Curr. Res. Food Sci..

[44] Li N., Chen F., Cui F., Sun W., Zhang J., Qian L., Yang Y., Wu D., Dong Y., Jiang J., Yang H. (2017). Improved postharvest quality and respiratory activity of straw mushroom (Volvariella volvacea) with ultrasound treatment and controlled relative humidity. Sci. Hortic..

[45] Aday M.S., Caner C. (2014). Individual and combined effects of ultrasound, ozone and chlorine dioxide on strawberry storage life, LWT. Food Sci. Technol..

[46] Jiang T., Feng L., Wang Y. (2013). Effect of alginate/nano-ag coating on microbial and physicochemical characteristics of shiitake mushroom (*Lentinus edodes*) during cold storage. Food Chem..

[47] Feng Y., Suo K., Zhang Y., Yang Z., Zhou C., Shi L., Chen W., Wang J., Wang C., Zheng Y. (2024). Ultrasound synergistic slightly acidic electrolyzed water treatment of grapes: impacts on microbial loads, wettability, and postharvest storage quality. Ultrason. Sonochem..

[48] Olotu I.O., Obadina A.O., Sobukola O.P., Adegunwa M., Adebowale A.A., Kajihausa E., Sanni L.O., Asagbra Y., Ashiru B., Keith T. (2015). Effect of chemical preservatives on shelf life of mushroom (*Pleurotus ostreatus*) cultivated on cassava peels. Int. J. Food Sci. Technol..

[49] Zheng Y., Zhu Y., Zheng Y., Hu J., Chen J., Deng S. (2022). The effect of dielectric barrier discharge plasma gas and plasma-activated water on the physicochemical changes in button mushrooms (Agaricus bisporus). Foods.

[50] Zhong Y., Cui Y., Wang X., Cong J., Yu J., Yan S., Bai J., Xu H., Li M. (2024). Electron-beam generated x-ray irradiation could retard the senescence of postharvest *Hericium erinaceus* via regulating reactive oxygen metabolism. Food Biosci..

[51] Ali M., Cheng J., Tazeddinova D., Aadil R.M., Zeng X., Goksen G., Lorenzo J.M., Esua O.J., Manzoor M.F. (2023). Effect of plasma-activated water and buffer solution combined with ultrasound on fungicide degradation and quality of cherry tomato during storage. Ultrason. Sonochem..

[52] Pourbagher R., Abbaspour-Fard M.H., Sohbatzadeh F., Rohani A. (2021). Inhibition of enzymes and *Pseudomonas tolaasii* growth on Agaricus bisporus following treatment with surface dielectric barrier discharge plasma. Innov. Food Sci. Emerg. Technol..

[53] Du Y., Mi S., Wang H., Yuan S., Yang F., Yu H., Xie Y., Guo Y., Cheng Y., Yao W. (2024). Intervention mechanisms of cold plasma pretreatment on the quality, antioxidants and reactive oxygen metabolism of fresh wolfberries during storage. Food Chem..

[54] Seelarat W., Sangwanna S., Panklai T., Chaosuan N., Bootchanont A., Wattanawikkam C., Subcharoen A., Subcharoen N., Chanchula N., Boonyawan D., Porjai P. (2023). Enhanced fruiting body production and bioactive phytochemicals from white cordyceps militaris by blending *cordyceps militaris* and using cold plasma jet. Plasma Chem. Plasma Process..

[55] Sun J., Fan J., Tong L., Ren R., Yao L., Wang D., Gu S. (2025). Combination effects of ultrasound and hyperbranched poly-l-lysine on quality preservation of fresh‑cut carrots during storage. J. Food Meas. Charact..

[56] Hu Y., Chen C., Xu L., Cui Y., Yu X., Gao H., Wang Q., Liu K., Shi Y., Chen Q. (2015). Postharvest application of 4-methoxy cinnamic acid for extending the shelf life of mushroom (Agaricus bisporus). Postharvest Biol. Technol..

[57] Cong K., Li T., Wu C., Zeng K., Zhang J., Fan G., Pan Y., Wang J., Suo A. (2022). Effects of plasma-activated water on overall quality of fresh goji berries during storage. Sci. Hortic..

[58] Ni J., Luo S., Bi Y., Zielinska S., Ding C., Tao J., Ning Z., Tian W., Peng W., Fang X. (2024). The combined effects of ultrasound and plasma-activated water on silkworm pupae:physicochemical properties, microbiological diversity and ultrastructure. Ultrason. Sonochem..

[59] Zhang T., Zhang Q., Lei Y., Yu X., Jiang H. (2023). Plasma activated water on improving the quality of fresh-cut banana slices. Postharvest Biol. Technol..

[60] Shi D., Yin C., Fan X., Yao F., Qiao Y., Xue S., Lu Q., Feng C., Meng J., Gao H. (2022). Effects of ultrasound and gamma irradiation on quality maintenance of fresh *Lentinula edodes* during cold storage. Food Chem..

[61] Wen X., Brunton N.P., Lyng J.G., Harrison S.M., Carpes S.T., Papoutsis K. (2020). Volatile and non-volatile compounds of shiitake mushrooms treated with pulsed light after twenty-four hour storage at different conditions. Food Biosci..

